# Cost analysis of technological vs. conventional upper limb rehabilitation for patients with neurological disorders: an Italian real-world data case study

**DOI:** 10.3389/fpubh.2024.1445099

**Published:** 2024-10-14

**Authors:** Valerio Gower, Irene Aprile, Francesca Falchini, Alessio Fasano, Marco Germanotta, Mattia Randazzo, Federico Spinelli, Leopoldo Trieste, Furio Gramatica, Giuseppe Turchetti

**Affiliations:** ^1^IRCCS Fondazione Don Carlo Gnocchi, Milan, Italy; ^2^IRCCS Fondazione Don Carlo Gnocchi, Florence, Italy; ^3^Institute of Management, Scuola Superiore Sant'Anna, Pisa, Italy

**Keywords:** cost analysis, robotic rehabilitation, cost minimization, probabilistic simulation, sensitivity analysis

## Abstract

**Introduction:**

Most patients suffering from neurological disorders endure varying degrees of upper limb dysfunction, limiting their everyday activities, with only a limited number regaining full arm use. Robotic and technological rehabilitation has been demonstrated to be a feasible solution to guarantee an effective rehabilitation to recover upper limb performance or to prevent complications of upper limb immobility. However, there is currently a lack of studies which analyze the sustainability of robotic and technological rehabilitation by comparing its costs to conventional rehabilitation pathways.

**Methods:**

Since technology-based and conventional rehabilitation of the upper limb have been demonstrated to have comparable efficacy when the rehabilitation dose is matched, our study concentrates on a cost minimization analysis. The aim of the study is to compare the costs of a “mixed” rehabilitation cycle, which combines conventional and technology-based treatments (the latter delivered with a single therapist supervising several patients), with a cycle of purely conventional treatments. This has been done by developing a cost model and retrospectively analyzing the costs sustained by an Italian hospital which has adopted such a mixed model. A sensitivity analysis has been done to identify the parameters of the model that have the greatest influence on cost difference and to evaluate their optimal values in terms of efficiency of mixed rehabilitation. Finally, probabilistic simulations have been applied to consider the variability of model parameters around such optimized values and evaluate the probability of achieving a given level of savings.

**Results:**

We found a cost difference of 49.60 € per cycle in favor of mixed rehabilitation. The sensitivity analysis demonstrated that, in the situation of the hospital under investigation, the parameter having the largest influence on the cost difference is the number of robotic treatments in a mixed rehab cycle. Probabilistic simulations indicate a probability higher than 98% of an optimized mixed rehabilitation cycle being less expensive than a pure conventional one.

**Conclusion:**

Through a retrospective cost analysis, we found that the technology-based mixed rehabilitation approach, within a specific organizational model allowing a single physiotherapist to supervise up to four patients concurrently, allowed cost savings compared to the conventional rehabilitation model.

## 1 Introduction

According to the 2019 Global Burden of Disease study (1), it is estimated that more than 255 million people suffering from neurological diseases require suitable rehabilitation therapy. Most patients suffering from neurological disorders (such as Stroke, Multiple Sclerosis, and Parkinson) experience varying degrees of upper limb disability, and only a limited number of those patients recover complete arm functions (2–5). As a result, most patients experience impairment and limitation in their ADL (Activities of Daily Living) due to upper limb dysfunction. Upper limb rehabilitation is very difficult both for the complexity of its function in the proximal segment (considering the numerous degrees of freedom of the shoulder joint) and for the precision of the hand movements. Therefore, the rehabilitation of the upper limb is an important challenge that often requires long-term treatment.

The International Classification of Functioning, Disability and Health model (6) defines upper limb disability as (i) impairments of body function, such as a significant deviation or loss in neuromusculoskeletal and movement-related function related to joint mobility, muscle power, muscle tone, and/or involuntary movements, or (ii) impairments of body structures, such as a significant deviation in the structure of the nervous system or structures related to movement.

In recent years, robotic and technological rehabilitation has emerged as one of the most promising approaches for restoring upper limb motor function after brain damage (7–10). Indeed, compared to conventional treatment approaches, it allows highly intensive training in specifically designed tasks over extended periods (11–13). Robotic therapy has been presented as a potential technique for upper limb rehabilitation, as a way to standardize treatment (14) and improve the volume and intensity of therapy, by offering complicated but regulated multimodal stimulation (15). After a stroke, for example, upper limb robotic therapy has been proven to enhance activities of daily living, arm function, and paretic arm muscular strength (16). In the context of large randomized controlled multicenter studies, robotics has been demonstrated to have at least a similar efficacy on upper limb recovery as conventional therapy administered in the same amount of time and number of sessions (17). This clinical effectiveness is linked to the mentioned possibility of intensive and highly controlled training, and to the ability to provide a motivating context through exergames (18).

Nonetheless, there are several factors that limit the widespread use of robotic systems in rehabilitation settings (19). Among these, the deployment of robots in healthcare settings is a challenging issue in terms of ensuring a sustainable environment. Robotic devices lie, in fact, in the highest range of costs among healthcare technologies. These costs are not limited to initial purchase costs, due to advanced hardware components and sensors and to sophisticated software, but involve also operating costs, including maintenance and energy consumption, and training costs for guaranteeing their adequate utilization by the clinical personnel. In an era of strong emphasis on healthcare resource allocation, there is a growing interest in reducing costs while maintaining a high-level quality of care. According to this perspective, economic assessments of innovative rehabilitation therapies are required (17, 20). These assessments have the potential to be a valuable tool for deciding on ways to translate research results into clinical practice, management, or health policy (21). Rigorous economic analyses may indeed support the advancement of cost-effective and sustainable rehabilitation strategies, ultimately enhancing patient outcomes and resource utilization in clinical settings.

There are, however, few studies in the rehabilitation field that look at the long-term viability of technological rehabilitation by analyzing its costs compared with conventional therapy. Wagner et al. (22) tracked stroke patients during the 36-week rehabilitation pathway and highlighted that the expenses of extra upper limb therapy due to robotic or intensive comparison therapy can be compensated by lower overall healthcare use costs with respect to usual care. More recently, Fernandez-Garcia and colleagues (23) performed a within-trial analysis of the cost-effectiveness of the RATULS trial (24), and revealed that neither robot-assisted training nor enhanced upper limb therapy (conventional rehabilitation focused on the upper limb rehabilitation), with a therapist-to-patient ratio of one to one, were likely to be cost-effective at any cost per Quality Adjusted Life Year levels considered. In this case, however, the economic evaluation was performed on a single patient’s population and based on a single rehabilitation cycle, in the trial scope. Thus, there is currently a substantial controversy and consequent skepticism around the potential benefit in terms of cost utility or cost-effectiveness in the large-scale deployment of advanced technology for the rehabilitation of people with neurological disorders (25).

On the other hand, it is crucial to point out that the organizational model adopted for the rehabilitation service is a determinant factor. A well-defined organizational model dictates how rehabilitation services are structured and delivered, thus playing a critical role not only in maximizing treatment efficacy but also in optimizing resource allocation and consequently ensuring accessibility for a wider patient population. In this sense, robotics and technological devices provide not just for increased treatment intensity but also for the treatment of more patients under the supervision of a single experienced therapist, hence boosting therapeutic efficiency and accessibility (26).

Indeed, according to Masiero et al. (27), the availability of a room equipped with more than one device might enhance the sustainability of the therapy, addressing an important requirement of under-resourced healthcare systems (28). In our previous feasibility study (29), we created and outfitted a robotic rehabilitation area with a set of four robotic and technological devices, capable of enabling a complete and tailored rehabilitation of the upper limb (30). In that study, we assessed the viability, in terms of rehabilitation dose and patient’s satisfaction, of a novel organizational model of robotic rehabilitation in which a group of up to four patients is monitored by a single physiotherapist, based on the severity of the impairment and the presence of comorbidities in each stage of upper limb robotic rehabilitation. Notably, even when supervising up to four patients concurrently, there was no reduction in the quality or intensity of therapy provided to individual patients. This underscores the potential of optimizing human resources while ensuring the delivery of effective rehabilitation interventions. In light of its positive clinical results demonstrated in a multicenter study (31), the organizational model of one therapist supervising three patients has been adopted as the standard clinical practice of the hospital *Santa Maria della Provvidenza* (SMP) of Fondazione don Carlo Gnocchi in Rome.

Given the current lack of studies investigating the long-term economic impact for healthcare providers of structured robotic rehabilitation in the clinical practice, the aim of this work was to assess the economic feasibility of our organizational model based on the robotic area within the clinical setting and the routine care of SMP hospital. Some studies and systematic reviews have shown that upper limb robotic training is just as effective as conventional training for patients with neurological disorders, when the rehabilitation dose is matched (17, 32). This equivalence is further confirmed by the results of a multicenter randomized clinical trial on upper limb robotic rehabilitation in stroke patients, conducted with the same set of devices under investigation in the current study; such trial found no significant differences in the improvement of Fugl-Meyer Assessment scores between robotic and conventional treatments (31). In light of the equivalent effectiveness between robotic and conventional rehabilitation, our study concentrates on a cost minimization analysis. In particular, a retrospective analysis was conducted with two main objectives: (i) to compare the costs incurred by this center in a specific timeframe for delivering upper limb treatments through mixed (i.e., integrated robotic and conventional) therapies with those hypothetically incurred if all treatments were conventional; (ii) to identify key parameters influencing the cost differentials, informing strategies to optimize the economic efficiency of robotic rehabilitation within the hospital framework. By elucidating the economic feasibility of the implemented rehabilitation approach, our study contributes valuable insights to healthcare decision-makers, enabling informed choices regarding the integration of robotic technologies into rehabilitation practices.

## 2 Materials and methods

### 2.1 Target population

In this study, we retrospectively analyzed the costs of neuromotor rehabilitation cycles, delivered with the support of four different technological devices that allow the rehabilitation of the entire upper limb for patients with neurological disorders. The target population includes primarily post-stroke, multiple sclerosis and Parkinson patients, accounting for 71, 10 and 5%, respectively. The remaining 14% is represented by patient with other neurological disorders including spinal cord injuries (whether traumatic, infection-related, or due to inflammation), different forms of polyneuropathy, amyotrophic lateral sclerosis, or other brain conditions such as hydrocephalus, Wilson’s disease, or brain tumors. The age ranges from a minimum of 18 years to a maximum of 93 years (median value 69 years, IQR 20.5 years). The percentage of female is around 45%.

### 2.2 Data collection process

At the SMP hospital of Fondazione Don Carlo Gnocchi in Rome, most of the neuromotor rehabilitation cycles of treatments are delivered following a *mixed* approach which combines conventional rehab sessions with technology-based ones. We considered for the analysis the costs of all the *mixed* neuromotor rehabilitation cycles (i.e., the ones that included at least one treatment delivered with robotic devices) delivered to patients belonging to three different settings: outpatients, inpatients and day cases.

We collected the data of all the *mixed* neuromotor rehabilitation cycles delivered at the SMP hospital during a three-year period ranging from January 2017 to December 2019. At the date of the data retrieval process, this was the longest period of available data which excludes the initial “transient” of technology adoption (robotic devices were acquired at the end of 2015) and the dramatic drop of treatments that occurred during the COVID pandemic situation from February 2020. The exclusion of those periods, where the number of treatments were significantly lower, allows us to evaluate the costs of rehabilitation cycles delivered by a “fully operational” hospital.

Direct costs of rehab treatments have been estimated based upon real data acquired by researchers from different sources. From the hospital economic department we retrieved the cost of devices and the annual cost of their maintenance, the average hourly costs of physiotherapists and assistants, the robot expected lifetime and the average cost of energy (€/kWh). The hospital economic department also provided the information to estimate the indirect costs (overheads) as a percentage of direct costs, as described into more details below. From the databases of the hospital information system we retrieved the list of rehab cycles delivered in the period under investigation including some information on the patient (sex, age, disease condition), the number of conventional and robotic treatment delivered, and the information on the setting in which treatments were delivered (inpatient, outpatient or day cases).

### 2.3 Hospital organizational models for technology-based and conventional rehabilitation

The organizational model of *robotic rehabilitation treatments* adopted by the hospital foresees a therapist that supervises a group of patients each interacting individually with a single device, as described into more details in (33). The number of patients supervised by a single therapist varies based on the severity of their clinical conditions. In particular, for outpatients, who are on average less severe, a single therapist supervises four patients at the same time, while for inpatients and day cases, who are more severe, three patients are supervised by a therapist. On the other hand, *conventional rehab treatments* are delivered with a 1 to 1 therapist-patient ratio and do not use any technological device.

### 2.4 Technological devices setup

The devices used in the technology based organizational model focus on specific upper limb districts: (1) MOTORE is a planar robot that enables shoulder and elbow movements in active, passive, and active-assisted modes. (2) Amadeo is a robotic device designed for hand rehabilitation that allows independent flexion-extension of individual fingers. (3) Pablo is a sensor-based device that enables unilateral and bilateral rehabilitation of the upper limb. (4) Diego is an electromechanical device designed for upper limb rehabilitation through a weight-relieving system. This set of devices, capable of providing a comprehensive rehabilitation of the upper limb, has been selected using a structured Health Technology Assessment procedure (29). Moreover, depending on their walking ability, some patients need the assistance of an operator (*assistant* in the following) to be transported to the gym. In the SMP hospital, outpatients are able to reach the robotic gym autonomously, while half of the day cases patients and all of the inpatients need the assistant.

### 2.5 Comparators

In our study we compared the costs of two alternatives: (i) the average cost sustained by the hospital for delivering a *mixed* cycle of rehabilitation treatments (i.e., the average cost of cycles that include at least one technology-based treatment) and (ii) the cost of a *conventional* rehabilitation cycle, including an equivalent average number of treatments, delivered without any support of technological devices. In the following the cost of a mixed rehabilitation cycle will be indicated with $C_{\mathrm{mix}}$ while the cost of a conventional rehabilitation cycle will be indicated with $C_{\mathrm{conv}}$ .

Both for mixed and conventional rehabilitation cycles, the duration of treatments is 45 min, the average number of treatments in a cycle is around 68, the average duration of a cycles is around 8.6 weeks.

As described above, in the technology-based treatments a single therapist supervises a group of patients composed of 4 (in the outpatient setting) or 3 (in the inpatient and day cases settings) subjects each interacting individually with a device. In the conventional therapy a single physiotherapist provides the treatment to a single patient.

### 2.6 Cost models and assumptions

In our study, we took the perspective of the healthcare provider, and therefore we evaluated the costs sustained by the hospital for delivering rehabilitation treatments. Direct costs of rehab treatments have been estimated based upon real data acquired from the hospital economic department and retrieved from the hospital information system. Based on data from the hospital’s economic department, the annual indirect costs (C_ind_) have been calculated by subtracting from the overall annual costs sustained by the hospital for rehabilitation (C_tot_) those budgetary items that were already considered as direct costs (e.g., costs of physiotherapists). This allowed us to estimate the indirect cost of rehabilitation cycles as the 25% of direct costs as follows: $ind\%=C_{ind}/\left(C_{\mathrm{tot}}-C_{ind}\right)\text{.}$

Based on the organizational models described above, a cost model has been defined to evaluate the cost difference between a *mixed* and a conventional rehabilitation cycle:


(1) $$\mathrm{DCost}=C_{\mathrm{mix}}-C_{\mathrm{conv}}$$

from Equation 1, it is trivial that the mixed rehab cycle costs less if DCost<0 .

The cost of a conventional rehabilitation cycle ( $C_{\mathrm{conv}}$ ) can be easily computed as shown in Equation 2:


(2) $$C_{\mathrm{conv}}=S\cdot C_{T}=S\cdot C_{\mathrm{ph}}\cdot T_{\mathrm{ph}}$$

where $C_{T}$ is the cost of a single conventional therapy treatment (in €) and S is the number of treatments in a rehabilitation cycle. $C_{T}$ is in turn calculated as the product of the hourly cost of the physiotherapist $C_{\mathrm{ph}}$ for the duration of the treatment $T_{\mathrm{ph}}$ .

Differently, $C_{\mathrm{mix}}$ is given by Equation 3:


(3) $$C_{\mathrm{mix}}=T\cdot C_{T}+R\cdot C_{r}$$

where T and R are, respectively, the number of conventional and robotic treatments in a cycle (thus, S=T+R ) and $C_{r}$ is the cost of a single robotic treatment. $C_{r}$ is calculated as the sum of the costs related to personnel (physiotherapist and assistant), robotic device depreciation, energy consumption and consumables (see Equations 4–11). To reflect the organizational model, the cost related to the assistant is considered as a weighted average of the costs in the three different settings (outpatients, day cases and inpatients). Similarly, the number of patients supervised by a single physiotherapist $P_{hn}$ is considered as a weighted average of the two different conditions (outpatients on the one side, and day cases + inpatients on the other), where the weight is the percentage of outpatients or day cases and inpatients cycles. A linear depreciation model has been used to evaluate the cost related to the equipment per single treatment (see Equation 6). We chose this linear model because, in the time-period analyzed, the number of annual treatments remains almost constant over the years. Moreover, the linear depreciation model is the one usually adopted by the economic department of the hospital to value its assets.


(4) $$C_{r}=C_{phs}+C_{pns}\cdot \left(1-P_{\mathrm{treat}\%}-\frac{H_{\mathrm{treat}\%}}{2}\right)+C_{\mathrm{depr}}+C_{e}+C_{\mathrm{cons}}$$

(5) $$C_{phs}=\frac{T_{\mathrm{ph}}\cdot C_{\mathrm{ph}}}{P_{hn}}$$

(6) $$C_{\mathrm{depr}}=\frac{p_{r}+m_{r}+C_{\mathrm{training}}}{lf_{r}\cdot R\cdot n}$$

(7) $$C_{e}=pe\cdot e\cdot T_{\mathrm{ph}}$$

(8–10) $$P_{\mathrm{treat}\%}=\frac{P}{n};H_{\mathrm{treat}\%}=\frac{H}{n};n=P+H+D$$

(11) $$P_{hn}=P_{hnp}\cdot P_{\mathrm{treat}\%}+P_{hnh-d}\cdot \left(1-P_{\mathrm{treat}\%}\right)$$

The meaning of all the terms of the cost model are reported in Table 1.

**Table 1:** List of all the terms and parameters in the cost model with their explanation.

Term	Explanation
DCost	Average cost difference between a mixed and a conventional rehab cycle (€/cycle)
C_conv_	Cost of a conventional rehab cycle (€/cycle)
C_mix_	Cost of a mixed (conventional+robotic) rehab cycle (€/cycle)
S	Average number of rehab treatments per cycle
T	Average number of conventional treatments in a rehab cycle
R	Average number of robotic treatments in a rehab cycle
C_T_	Cost of a single conventional therapy treatment (€/treatment)
C_r_	Cost of a single robotic therapy treatment (€/treatment)
C_phs_	Cost of physiotherapist per robotic treatment (€/treatment)
C_pns_	Cost of assistant per robotic treatment (€/treatment)
P_treat%_	Percentage of P (outpatients) rehab cycles over the total number of rehab cycles
H_treat%_	Percentage of H (day cases) rehab cycles over the total number of rehab cycles
P	Average number of outpatients’ rehab cycles per year
D	Average number of inpatients’ rehab cycles per year
H	Average number of day cases’ rehab cycles per year
C_depr_	Cost of robot depreciation per treatment (€/treatment)
C_e_	Cost of energy per treatment (€/treatment)
C_cons_	Cost of consumables per treatment (€/treatment)
T_ph_	Duration of a treatment (h)
C_ph_	Hourly cost of physiotherapist (€/h)
P_hn_	Number of patients supervised by a physiotherapist (weighted average)
P_hnp_	Number of patients supervised by a physiotherapist in outpatients setting
P_hnh-d_	Number of patients supervised by a physiotherapist in inpatients and day cases settings
p_r_	Price of robotic solution (including all 4 robots) (€)
m_r_	Cost of robot maintenance for the entire lifetime (€)
C_training_	Cost of personnel training (€)
lf_r_	Expected lifetime of robotic solution (years)
n	Average number of rehab cycles per year
pe	Price of a kWh (€)
e	Average power absorption of the robotic solution (kW)

In our model, we considered the set of 4 devices as a single robotic solution since all of them are needed to target all upper limb functionalities during a rehabilitation cycle.

### 2.7 Sensitivity analysis

To evaluate the sensitivity of the model to its parameters, we used the method of partial derivatives (34): we computed the analytical formulas of partial derivatives and then evaluated their numeric values in a given point of the n-dimensional space of parameters. In particular, we evaluated the partial derivatives in the “situation” of the SMP hospital by entering in the formulas the average values of parameters over the considered 3 years period. The final aim of this analysis is to identify the set of parameters on which the healthcare provider should act to reduce the costs of robotic rehabilitation in the most effective way. Therefore, in this sensitivity analysis, we considered fixed those parameters that are not under the control of the healthcare provider, in particular: the cost of robotic solutions, maintenance and training, cost of energy consumption and consumables, cost of personnel, duration of treatments, and expected lifetime of robots. We also considered fixed the parameters related to the organizational model and in particular the number of patients supervised by a single physiotherapist. The parameters left to vary were therefore R (number of robotic treatments in a rehab cycle), *P* (number of outpatients’ rehab cycles per year), *D* (number of inpatients’ rehab cycles per year) and *H* (number of day cases’ rehab cycles per year).

In consideration of the fact that the values of the four parameters (*R*, *P*, H and *D*) recorded in the SMP hospital have different magnitudes, we also computed the Differential Importance Measure (*DIM*) of such parameters. Following the method of Borgonovo et al. (35) DIM is computed starting from Elasticity (*E*) as shown in Equations 12 and 13.


(12) $$DIM_{i}=E_{i}/\overset{n}{\underset{j=1}{\sum }}E_{j}$$

(13) $$E_{i}=\frac{\partial g\left(x^{0}\right)}{\partial x_{i}}\;\cdot \;\frac{x_{i}^{0}}{g\left(x^{0}\right)}$$

Where $g\left(x\right)$ is the function of the model under study (in our case *DCost* as a function of *R*, *P*, H and *D*), xi are the parameters of the model (in our case *R*, *P*, H and *D*) and $x_{i}^{0}$ is reference value of the *i^th^* parameter (in our case the values of parameters in the situation of SMP hospital).

Differently from the partial derivatives method, which allows to evaluate the impact of parameters’ change under the assumption of uniform perturbations (i.e., unitary increment of parameters), *DIM* allows to evaluate the same impact under the assumption of proportional perturbations (i.e., variations of parameters in percentage of their values).

We then took the parameter with highest influence (i.e., the one with the highest absolute value of partial derivatives in the considered point of the n-dimensional parameter space) and identified value such parameter should assume to maximize the cost difference (*DCost*), given the following two constraints:

- Organizational constraint: maximum number of robotic treatments per year that can be delivered in the robotic gym ( R·n )
- Clinical constraint: maximum average percentage of robotic treatments in a cycle ( R/S ).

The latter is a constraint that derives from the fact that not all rehabilitation treatments can be delivered with the identified set of robots. In particular, most of the patients that undergo upper limb rehabilitation, also require lower limb treatments, which cannot be delivered with the set of robotic devices described in this paper.

### 2.8 Probabilistic simulation and statistical analysis

A probabilistic simulation approach has been used to take into account the intrinsic stochastic variability of some of the model parameters (36). In particular, in our model, we applied the stochastic variability to the parameters related to the number of rehab cycles delivered by the hospital in the different settings (*P, H* and *D*) and to the number of robotic (*R*) treatments in each cycle. For each of the four parameters (*P, H, D* and *R*) we estimated the Probabilistic Density Function (*PDF*) fitting the most common distributions to histograms of historical data recorded in the SMP hospital over the analyzed period. The estimation of the PDFs has been done with the fitdist (fitdistrplus library) method of the R software (37). In particular, for each one of the five parameters we fitted the following distributions: truncated normal, exponential, weibull and gamma. For the parameters P, D and H, to increase the number of available samples, the distributions have been fitted on the number of cycles per months (*P_month_, D_month_* and *H_month_*) instead of number of cycles per year. In the simulations, the values sampled on *P_month_, D_month_* and *H_month_* distributions have then been multiplied by 12 to obtain the number of cycles per year. We selected the different PDFs, one per parameter, that minimize the Akaike Information Criterion (AIC) figure of merit (38).

The selected PDFs were then used to run simulations aimed at evaluating the average cost difference between a mixed and a conventional rehabilitation cycle in a year (DCost). As a first step we generated 3 arrays of 130 values of the parameters P, H and D, by sampling on the respective PDFs, and calculated the corresponding 130 values of n:


(14) $$n_{i}=P_{i}+H_{i}+D_{i}\;\mathrm{with}\;i=1,\ldots ,130$$

The optimal number of samples (130) has been determined following the method proposed by Liu (39) by setting the confidence interval to 95% and the precision to 6 €.

For each of the 130 values of n (which represent 130 simulated years) we extracted n_i_ values of R from its PDF, and calculated the number of robotic treatments in the simulated year (RY, Equation 15) as the sum of all the extracted R:


(15) $$RY=\overset{n_{i}}{\underset{j=1}{\sum }}R_{j}$$

Finally, we calculated 130 average cost differences with Equation 16.


(16) $$\mathrm{DCos}t_{i}=\left({C_{r}}_{i}-C_{T}\right)\cdot \frac{1}{n_{i}}\cdot \overset{n_{i}}{\underset{j=1}{\sum }}R_{j}$$

In order to take into account the organizational constraint, a check was added to verify, at each iteration of *j,* if the partial sum of R_j_ $\left(\overset{k}{\underset{j=1}{\sum }}R_{j}\right)$ exceeded the threshold of the maximum number of robotic treatments that can be delivered in 1 year. When this happens, RY is set to the value of the threshold and n_i_ is set to the number of iterations reached before exceeding the threshold (n_i_ = k–1).

Finally, we calculated the probability p of saving at least a given value x ( $p\left(\mathrm{DCost}<x\right)$ ). To better estimate the value of p , we repeated the simulation until the difference between the mean of probabilities at iteration *h* and the mean of probabilities at iteration *h-1* was less than 10^−3^.

The hypothesis that patients included in the 3 different settings (P, H, and D) have significantly different severity level (based on the Barthel Index score at admission) was validated through a Kruskal-Wallis test between all groups and then a Wilcoxon post-hoc test with the Bonferroni correction.

All the statistical analysis and probabilistic simulation have been performed with the software R (version 4.3.2).

## 3 Results

**Figure 1:**
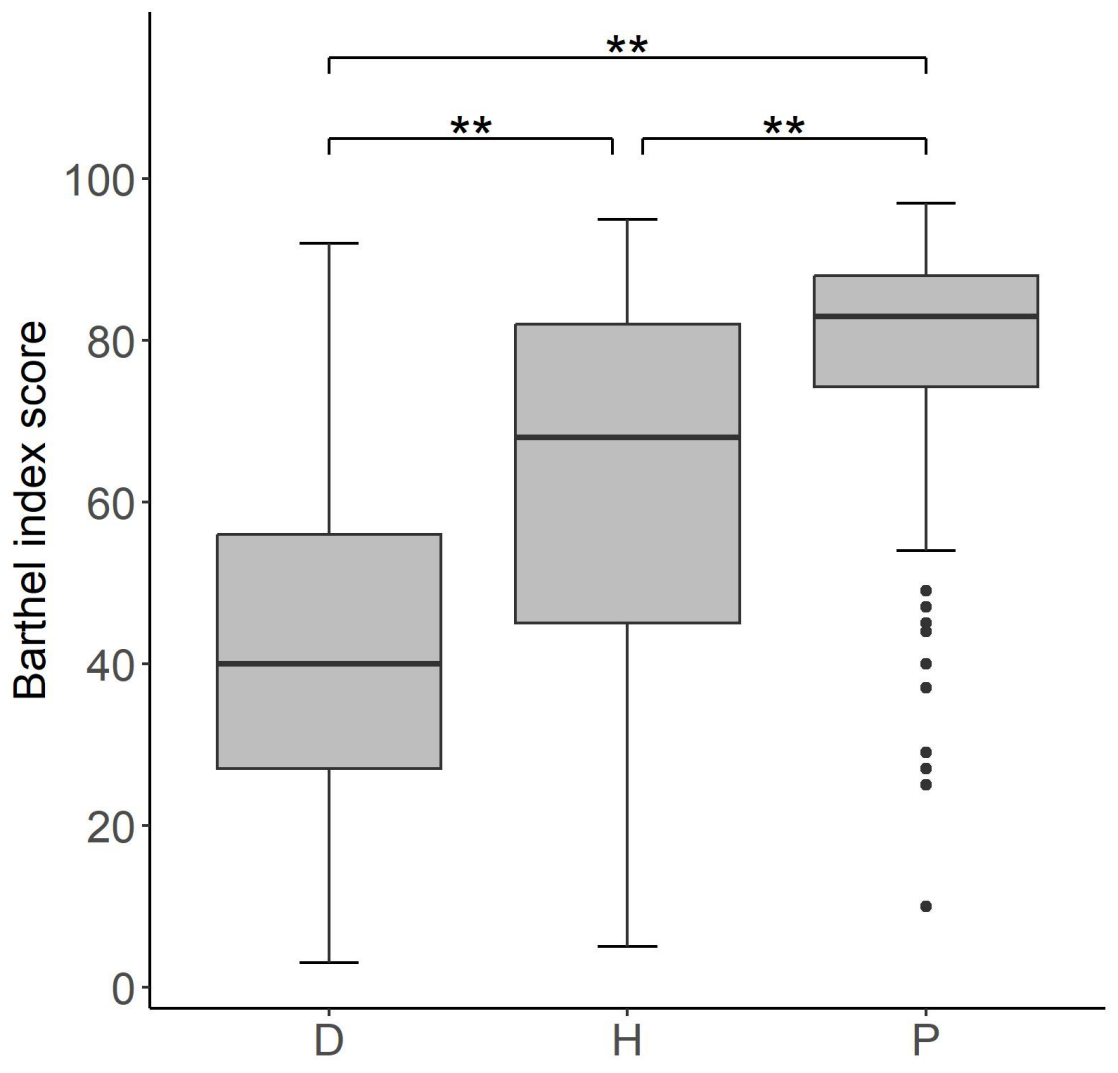
Box plot of Barthel index values at admission for the three different groups of patients D (inpatients), H (day cases) and P (outpatients). The lower and upper side of each box represent the first and third quartile, respectively. The horizontal bold line shows the median value. Whiskers extend to the most extreme data point which is no more (or less) than 1.5 times the interquartile range from the box, while circles represent outliers. Double asterisks indicate *p*-values <0.001.

Differences in clinical conditions of outpatients’, day cases’ and inpatients’ Barthel Index scores at admission are shown in Table 2. The *post-hoc* analysis has identified significant differences among the three groups (*p*-values <0,001 for all possible group pairs) in terms of impairment level (Figure 1).

**Table 2:** Values of Barthel index at admission for the three different settings (outpatients, day cases and inpatients).

Group	Median (q_1/2_)	First quartile(q_1/4_)	Third quartile (q_3/4_)
outpatient	83.00	74.25	88.00
day cases	68.00	45.00	82.00
inpatients	41.87	27.00	56.00

### 3.1 Data used for calculation

In the time period considered (i.e., between January 1st, 2017 and December 31st, 2019), 891 mixed rehabilitation cycles have been performed at the SMP hospital, including 60,534 neuromotor treatments in total, out of which 15,933, corresponding to 26.32%, were performed with upper limb robotic devices. Out of the 891 rehab cycles, 83 involved outpatients (P_treat%_ = 9.32%), 306 day-cases (H_treat%_ = 34.34%), and 502 inpatients (D_treat%_ = 56.34%). The average number of treatments per cycle (S) is 67.94, the average number of robotic treatments per cycle is 17.88 and the average number of conventional treatments per cycle is 50.06.

The average hourly costs sustained by the SMP hospital for physiotherapists and assistants in the considered period were 19.50 €/h and 16.48 €/h, respectively. The duration of a single treatment (both robotic and conventional) is 45 min.

For what concerns the costs related to energy consumption, we considered an average price of energy (pe) of 0.36 €/kWh and an average power absorption (e) of 0.28 kW, which is the average of the maximum power absorption of the four robots.

The overall purchase cost of the robotic solutions, which were acquired in the end of 2015, (p_r_) was 152,256 €, VAT included, while the maintenance costs (m_r_) were 15,767 € per year. In compliance with the standard accounting procedure of the hospital, the robotic solution lifetime (lf_r_) was considered to be 8 years.

With regard to training on the use of robotic solutions, this was initially provided by the manufacturers to a small group of physiotherapists without charging additional costs. This group of experienced physiotherapists was subsequently periodically involved in training their peers. For this reason, we evaluated the cost of training in terms of productivity loss. In particular, we estimated that 75 physiotherapists’ slots (each lasting 45 min) need to be devoted to training during the robot’s lifetime, resulting in an overall cost of 1371.09 €.

As described above, a percentage of 25% was added to all direct costs (both for conventional and robotic treatments) to account for facility overheads.

The maximum number of robotic treatments per year that can be delivered in the robotic gym (R*n) has been calculated based on the following data:

- The robotic gym is open 50 weeks a year,
- Each week, 55 rehab slots are available (10 slots per day from Monday to Friday plus 5 slots on Saturday),
- Three treatments can be delivered in each slot.

In total, 8,250 treatments/year can be delivered in the robotic gym.

On the other hand, based on the experience of SMP clinicians, the maximum average percentage of robotic treatments in a cycle (R/S) has been set to 35%.

### 3.2 Cost difference

All the data reported above have been used to evaluate the overall cost of mixed rehab cycles actually sustained by the hospital in the 3-years period and to calculate the theoretical cost of an equivalent number of conventional treatments. The difference (DCost) between the costs of the actual mixed rehab cycles delivered in the 3 years and the theoretical conventional equivalent is −44191.06€, in favor of mixed therapy. Considering the 891 mixed rehab cycles delivered in the period, this means an average saving of −49.60€ per mixed cycle compared to a conventional one.

The cost difference between a single robotic treatment and a conventional one (C_r_ - C_T_) is −2.77 € in favor of robotics, which represents a saving of 15.17% with respect to the cost of a conventional treatment.

### 3.3 Sensitivity analysis

The values reported in Table 3, representing the average values of the parameters recorded in the SMP hospital in the 3 years, have been used to calculate the numeric values of partial derivatives and DIMs.

**Table 3:** Average values of parameters R, P, D and H used for evaluating partial derivatives.

Parameter	Average value in the considered period
R	17.88 (treatments/cycle)
P	27.67 (cycles/year)
D	167.33 (cycles/year)
H	102.00 (cycles/year)

Evaluating the partial derivatives and DIMs in the positions of Table 3 results in the values reported in Table 4.

**Table 4:** Results of partial derivatives and DIM evaluated in the situation of SMP hospital.

Parameter	Partial derivative value	DIM
R	−10.53	0.576
P	−0.65	0.055
D	−0.43	0.219
H	−0.48	0.150

The DIM provides information on the relative importance of the model parameters, while the direction of change (i.e., if an increment of the parameter causes DCost to increase or decrease) is given by the corresponding partial derivative. Given the definition of DCost in Equation 1, the more negative the derivative of a parameter is, the less a robotic rehabilitation cycle costs for higher values of such parameter.

**Figure 2:**
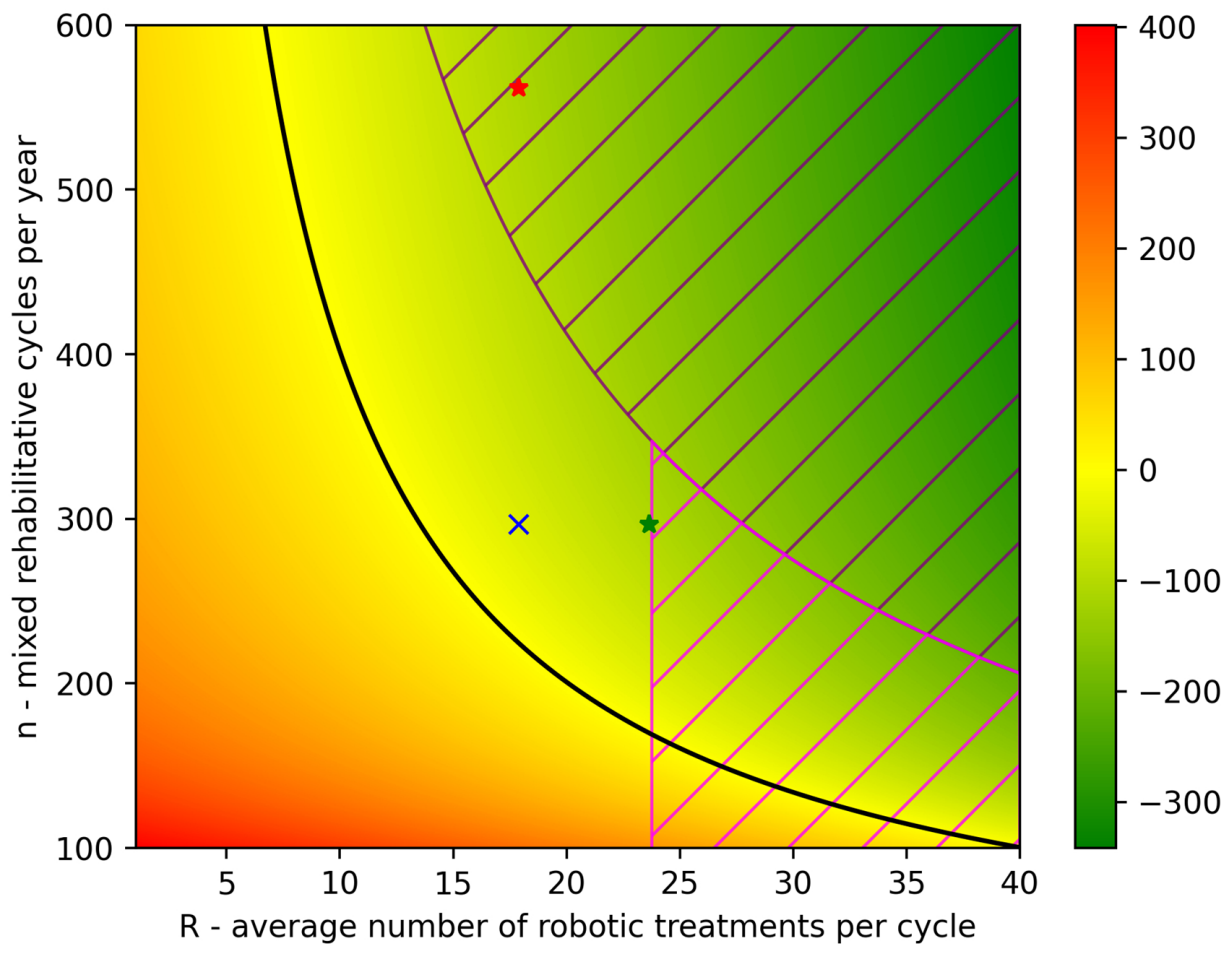
Color-map representing how DCost varies in function of the parameters R and n. The DCost gradient is represented on the right side of the figure and moves from green (DCost<0) to red (DCost>0). The black line represents the situation where DCost = 0. The situation of the SMP hospital is represented by the blue cross (*R* = 17.88 and *n* = 297). The dashed area represents the situations that cannot be achieved due to the organizational and/or clinical constraints ( R·n<=8250 and R/S = 0.35, where S = 67.94). The green star represents the maximum DCost achievable by increasing R while the red star represents the same DCost value achieved by increasing n.

Under the assumption that the proportions between P, D and H do not vary, we can evaluate the variation of DCost as a function of the two independent variables n (*n* = P + D + H) and R. The color-map in Figure 2 shows how DCost changes as the two parameters R and n vary. Negative values of DCost (i.e., in favor of mixed rehabilitation) are represented in green, positive values of DCost (i.e., in favor of conventional rehab) have a red color, and the black line represents the boundary between these two situations (i.e., DCost = 0). In the color-map presented in Figure 2, the SMP situation is represented by the blue cross (*R* = 17.88 and *n* = 297) that lies on the green area. Suppose now to be interested in maximizing the savings while respecting the organizational and clinical constraints (i.e., R·n<=8250 and R/S<=0.35 ).

By increasing the parameter R to the maximum value that respects the constraint R/S<=0.35 (i.e., R=0.35·67.94=23.78 ) it is possible to obtain a saving of 111.70 € in favor of a mixed rehabilitation (green star in Figure 2). Such value is therefore achievable with an increment of R of +32%. On the other hand, to achieve the same DCost value (red star in Figure 2), the parameter n would need to be increased by 77% to reach 562 cycles per year. However, the latter scenario cannot be implemented as the number of robotic treatments needed, exceeds those that the robotic gym can provide, according to the constraints described previously. Being DCost a monotonically decreasing function of both R and n, the maximum savings can be obtained by increasing both R and n in such a way that they reach the limits given by the constraints: *R* = 23.78 and *n* = 346.93. Consequently, the maximum possible value of DCost is −131.68 €.

### 3.4 Probabilistic simulation and statistical analysis

**Figure 3:**
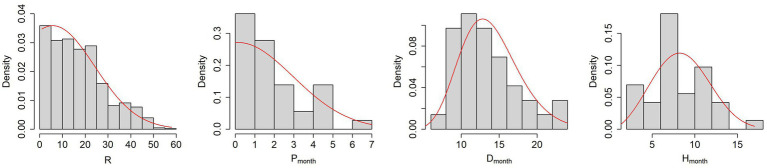
From left to right, histograms of the parameters R, P, D and H with the PDFs fitting obtained by minimizing the AIC (red lines).

Figure 3 and Table 5 show the results of the probability density distributions fitting obtained by minimizing the AIC.

**Table 5:** Results of distribution fitting procedure The name of the distributions and the parameters defining their shape are reported.

Parameter	Distribution name	Parameters
R	Truncated normal	Mean = 5.77; standard deviation = 18.48; left limit = 1
P_month_	Truncated normal	Mean = 0.13; standard deviation = 2.84; left limit = 0
H_month_	Gamma	Shape = 12.8; rate = 0.92
D_month_	Weibull	Shape = 2.88; scale = 9.54

It is worth noticing that the mean parameter of truncated normal distributions is not equal to the mean value of the sampled distribution.

Using the PDFs described in Table 5, we performed two different simulations.

The first one was aimed at evaluating the probability of obtaining given levels of savings when the average number of robotic treatments (R) is increased to 23.78. In particular, we evaluated the probability that the mixed rehabilitation costs less than the conventional one [i.e., $p\left(\mathrm{DCost}<0\right)$ ] and the probability of obtaining savings equal to at least 85% of −111.70€ [i.e., $p\left(\mathrm{DCost}<-94.95\right)$ ].

The second simulation was aimed at evaluating the probability of obtaining given levels of savings when both parameters R and n are increased to achieve the maximum possible level of saving (i.e., *R* = 23.78; *n* = 346.93; DCost = −131.68 €). Similarly, to the first simulation, we evaluated the probability that the mixed rehab cost less than the conventional one [i.e., $p\left(\mathrm{DCost}<0\right)$ ] and the probability of obtaining savings equal to at least 85% of 131.68€ [i.e., $p\left(\mathrm{DCost}<-111.93\right)$ ].

For the first simulation we sampled the values of the parameter R on a translation of its PDF obtained by increasing the value of the mean parameter in such a way to obtain a distribution whose sampled data had an average value of 23.78. The resulting translated PDF of R have a mean parameter value of 17.3. For the second simulation we also translated the PDF of n in such a way to obtain a distribution whose sample data had an average value of 346.93.

**Figure 4:**
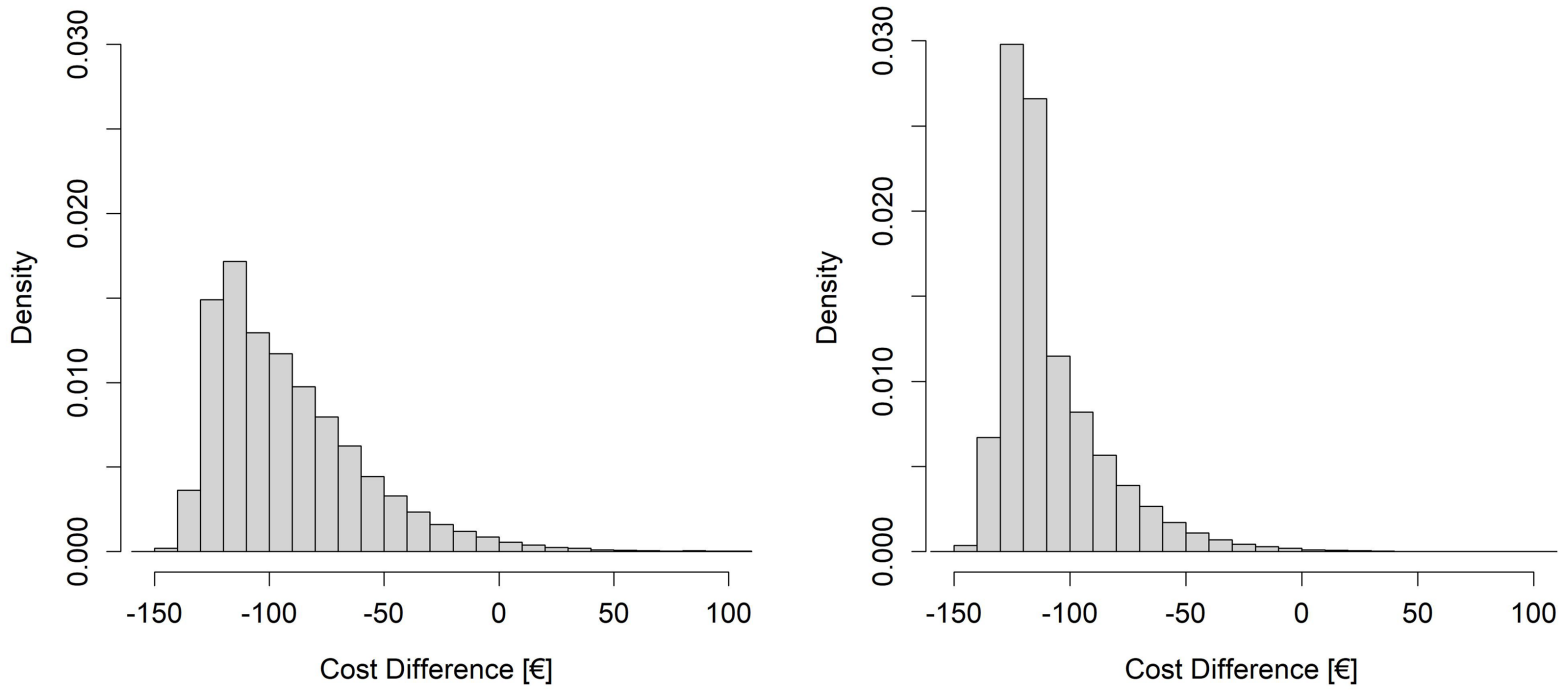
Histograms resulting from the simulations of cost differences (DCost). On the left side the simulation with R varying around the mean value of 23.78 and n fixed at 297, on the right side the simulation with R varying around the mean value of 23.78 and n varying around the mean value of 346.93.

Figure 4 depicts the histograms of the two simulations and Table 6 reports the values of probability in the situations described above.

**Table 6:** Results of the simulations The fourth column reports the probability of obtaining at least the levels of savings indicated in the third column, with the parameters R and n indicated in the first two columns.

R	n	DCost threshold	Probability of saving at least DCost threshold
23.78	297	<0 €	98.14%
23.78	297	<−94.95 €	79.5%
23.78	346.93	<0 €	99.59%
23.78	346.93	<−111.93 €	62.62%

## 4 Discussion

In this work, we presented a model that allows to calculate the cost difference between mixed (i.e., a combination of robotic and conventional treatments) and purely conventional rehabilitation. The model, retrospectively applied to the data retrieved from one of the hospitals of Fondazione don Gnocchi, showed that the mixed rehabilitation approach allowed to reduce the cost of rehabilitation compared to a conventional rehabilitation. By analyzing the sensitivity of the model to its parameters, it was possible to identify the conditions for optimizing the efficiency of robotic rehabilitation. Finally, the introduction of a probabilistic simulation approach allowed to take into consideration the intrinsic variability of the parameters of the model and evaluate the probability of achieving given level of savings with the robotic rehabilitation.

The model, the graphical representation of cost difference, and the probabilistic simulation approach are tools to support healthcare providers in making data-driven decisions about investing in robotic rehabilitation.

The statistically significant differences of the values of Barthel index at admission among the three groups of patients (inpatients, day cases and outpatients) support the organizational model adopted by the SMP hospital, which differentiate the rehabilitation procedures, and consequently the level of costs, among the three groups.

The mixed rehabilitation approach (i.e., a combination of robotic and conventional treatments in the rehabilitation cycle) adopted by the SMP hospital allowed to save more than 44 thousands € in a 3-years period. This represents a saving of around 4% with respect to the overall costs of conventional neuromotor rehabilitation, which includes both upper and lower limb treatments. If we focus on the upper limb treatments, which are the target of the technological devices considered and represent around 35% of the total neuromotor treatments delivered in the hospital, the mixed approach allowed to reduce the costs related to the rehabilitation of the upper limb district of around 11%.

The sensitivity analysis of the cost model clearly demonstrated that, in the situation of the SMP hospital, which already has a considerable number of mixed rehab cycles per year (n), the parameter having the largest influence on the cost difference is the number of robotic treatments per cycle (R) as demonstrated both by the partial derivatives and the DIM. This means that the efficiency of robotic rehabilitation in the SMP hospital can be improved more effectively by increasing the number of robotic treatments administered in each cycle, rather than, for example, recruiting new patients to increase the number of mixed rehabilitation cycles delivered. In our model, we also introduced two constraints that reflect the maximum number of robotic treatments that can be delivered in the robotic gym (8,250 per year—organizational constraint) and the maximum average percentage of robotic treatments in a rehab cycle (35%—clinical constraint). The latter takes into consideration the fact that not all the neuromotor rehab treatments can be delivered using the set of technological devices described in this paper. These two constraints must be taken into account when defining the values of the parameters that optimize the cost difference (DCost). In consideration of this limits, the highest level of savings that can be achieved by increasing only the R parameter (DCost_maxR_) is −111.70 € per cycle, which represent a saving of around 9% with respect to the overall costs of conventional neuromotor rehabilitation and 26% of the costs of upper limb treatments. On the other hand, a similar level of savings cannot be achieved by increasing the parameter n alone due to the constraint on the maximum number of robotic treatments that can be delivered each year. The same result is clearly represented by the color-map of Figure 2 that presents the situation of the SMP hospital (blue cross) and the increases that should be done on parameters R (green star) and n (red star) to achieve the same level of savings (−111.70 € per cycle).

The maximum level of saving, achievable by increasing at the same time R and n (DCost_max_) in such a way that they reach the limits given by the organizational and clinical constraints, is 131.68 € which represent a saving of around 10.6% with respect to the overall costs of conventional neuromotor rehabilitation and 30.3% of the costs of upper limb treatments.

In our work, we introduced a probabilistic simulation approach to take into account the intrinsic variability of some of the parameters of the model. In particular, the number of robotic treatments prescribed by a clinician to a specific patient (reflected in the parameter R) depends on a number of factors that cannot be easily predicted or modeled. A similar consideration holds true for the number of rehab cycles that the hospital performs each year in the different settings (reflected in the parameters P, H, and D) which depend on factors that are not fully under the control of the healthcare provider. The other parameters (e.g., cost of robots, cost of maintenance, cost of consumables, robot lifetime, hourly cost of personnel, and duration of treatment) can be considered less variable.

We applied the probabilistic simulation approach to evaluate the probability of achieving a given level of savings by varying the parameters R and n around the values that allow to achieve the maximum possible level of savings. Table 6 shows that simulations indicate a high probability (*p* > 98%) of achieving savings (DCost<0) when the average number of robotic treatments per cycle is 23.78 and the number of mixed rehabilitation cycles per year is at least 297. The probabilities of achieving at least 85% of the maximum savings achievable by increasing R only, or R and n together, are lower, as expected: $p\left(\mathrm{Dcost}<0.85\cdot \mathrm{DCos}t_{\mathrm{maxR}}\right)=79.5\%$ and $p\left(\mathrm{Dcost}<0.85\cdot \mathrm{DCos}t_{\text{max}}\right)=62.62\%$ . The difference between these two probabilities is due to the fact that, to achieve the maximum level of savings (DCost_max_), the robotic gym must be used close to its maximum capacity and, given the variability of R and n together with the organizational constraint (R*n < 8,250), this is less probable than achieving the lower number of robotic treatments per year required to reach DCost_maxR_.

The results of our study demonstrate that technology-based upper limb rehabilitation pathways can be less expensive, compared to conventional rehabilitation, if an appropriate organizational model is applied. This is in line with what was found in other studies where one physiotherapist supervised more than one patient at the same time (22, 27, 40). On the other hand, as one would expect, when a 1 to 1 patient-physiotherapist ratio is applied, technological rehabilitation results more expensive than the conventional one (23).

Compared to other studies which have analyzed the costs of upper limb robotic rehabilitation based on hypothetical number of yearly robotic treatments that could potentially be delivered with a given organizational model (22, 23, 27, 40), our approach is based on the retrospective analysis of real world data, and this allowed us to estimate the actual savings achieved by the hospital.

Our economic analysis must be understood in light of the fact that upper limb treatment necessitates a large number of rehabilitation sessions, which are frequently made possible by technological advancements. In fact, one of the goals of adopting rehabilitation technologies is to increase therapy intensity. This is critical considering the rehabilitation field’s ongoing human resource scarcity. As a result, increasing the number of robotic sessions in a single therapy cycle may represent not only a strategy to get better therapeutic results, but also to make the treatment more economically feasible.

The results of this study are expected to provide valuable insights for healthcare providers and policymakers. By demonstrating the economic feasibility of the multi-patient technological rehabilitation model, this research can contribute to the wider adoption of this innovative technology. This, in turn, has the potential to improve access to effective upper limb rehabilitation for a larger patient population.

## 5 Limitations

The study has some limitations to be acknowledged. Firstly, all the data is derived from a single hospital and this limits the generalizability of the findings to other settings or countries. Additionally, while the study primarily focuses on cost analysis, it does not provide details on the long-term clinical outcomes and the sustainability of robotic rehabilitation which should be further investigated.

The assessment of the study through the Consolidated Health Economic Evaluation Reporting Standards checklist (41) indicates that 23 items, out of the 28, are fulfilled. The missing items are relative to a formal document for the health economic analysis plan (item 4), the characterization of subgroups of the target population (item 18) and the consequent analysis of impact for the different subgroups (item 19), and the lack of engagement with patients for the design of the study (item 21) and consequently the missing analysis of impact of such involvement (item 25).

## 6 Conclusion

This study examined the economic feasibility of integrating technological and conventional therapies for upper limb rehabilitation in the clinical practice of an Italian rehabilitation hospital equipped with a robotic gym. Through a retrospective cost analysis, we found that the mixed rehabilitation approach, within a specific organizational model allowing a single physiotherapist to supervise up to four patients concurrently, allowed cost savings compared to the conventional rehabilitation model. These findings, together with the clinical effectiveness of the robotic approach, underscore the viability of leveraging technologies to optimize resource utilization and enhance rehabilitation outcomes, offering valuable insights for healthcare decision-makers in terms of the sustainability of robot-assisted rehabilitation practices in real clinical settings.

## Data Availability

The original contributions presented in the study are included in the article/supplementary material, further inquiries can be directed to the corresponding author/s.
